# Extent and Distribution of Parenchymal Abnormalities in Baseline CT-Scans Do Not Predict Awake Prone Positioning Response in COVID-19 Related ARDS

**DOI:** 10.3390/diagnostics12081848

**Published:** 2022-07-30

**Authors:** Federico Raimondi, Sara Cazzaniga, Simona Annibali, Luca Novelli, Matteo Brivio, Simone Pappacena, Luca Malandrino, Pietro Andrea Bonaffini, Ilaria Bianco, Noemi Liggeri, Paolo Gritti, Ferdinando Luca Lorini, Sandro Sironi, Fabiano Di Marco

**Affiliations:** 1 Pulmonary Medicine Unit, ASST Papa Giovanni XXIII, 24127 Bergamo, Italy; fraimondi@asst-pg23.it (F.R.); lnovelli@asst-pg23.it (L.N.); simone.pappacena@unimi.it (S.P.); luca.malandrino@unimi.it (L.M.); 2 Department of Intensive Critical Care, ASST Papa Giovanni XXIII, 24127 Bergamo, Italy; scazzaniga@asst-pg23.it (S.C.); mbrivio@asst-pg23.it (M.B.); pgritti@asst-pg23.it (P.G.); llorini@asst-pg23.it (F.L.L.); 3 Department of Diagnostic Radiology, ASST Papa Giovanni XXIII, 24127 Bergamo, Italy; sannibali@asst-pg23.it (S.A.); pbonaffini@asst-pg23.it (P.A.B.); ilaria.bianco.ib@gmail.com (I.B.); noemiliggeri@gmail.com (N.L.); ssironi@asst-pg23.it (S.S.); 4 Department of Medicine and Surgery, University of Milano-Bicocca, 20126 Milan, Italy; 5 Department of Health Sciences, University of Milan, 20122 Milan, Italy

**Keywords:** COVID-19, CT, ARDS, respiratory failure, mortality, comorbidities

## Abstract

Prone positioning is frequently used for non-intubated hypoxemic patients with COVID-19, although conclusive evidence is still lacking. The aim of the present study was to investigate whether baseline CT-scans could predict the improvement in oxygenation in COVID-19 related Acute respira-tory syndrome (ARDS) patients when pronated. Methods: A retrospective study of COVID-19 patients who underwent non-invasive ventilation (NIV) and prone positioning was conducted. Results: Forty-five patients were included. On average, 50% of the overall lung volume was affected by the disease, as observed in the CT-scans, with ground glass opacities (GGOs) and consolidations accounting for 44% and 4%, respectively. The abnormalities were mainly posterior, as demonstrated by posterior/anterior distribution ratios of 1.5 and 4.4 for GGO and consolidation, respectively. The median PaO_2_/FiO_2_ ratio during NIV in a supine position (SP1) was 140 [IQR 108–169], which improved by 67% (+98) during prone positioning, on average. Once supine positioning was resumed (SP2), the improvement in oxygenation was maintained in 28 patients (62% of the overall population, categorized as “responders”). We found no significant differences between responders and non-responders in terms of the extent (*p* = 0.92) and the distribution of parenchymal abnormalities seen in the baseline CT (*p* = 0.526). Conclusion: Despite the lack of a priori estimation of the sample size, considering the absence of any trends in the differences and correlations, we can reasonably conclude that the baseline chest CT-scan does not predict a gas-exchange response in awake prone-positioned patients with COVID-19 related ARDS. Physicians dealing with this category of patients should not rely on the imaging at presentation when evaluating whether to pronate patients.

## 1. Introduction

The coronavirus disease 2019 (COVID-19) pandemic led to a dramatic increase in the number of patients who developed pneumonia and acute respiratory failure, requiring respiratory support [1]. Acute respiratory distress syndrome (ARDS) is a serious complication of COVID-19 that can affect up to 40% of hospitalized patients, resulting in a high dependency unit and intensive care unit (ICU) overload [2,3]. Therefore, clinicians have been led toward exploring new approaches in order to limit the need for invasive mechanical ventilation, including awake prone positioning (i.e., in non-intubated patients) [4]. In intubated patients with moderate to severe ARDS that is not due to COVID-19 prone positioning, this leads to an optimization of oxygenation and a reduction in mortality [5]. The benefit of oxygenation is primarily attributed to a more homogeneous ventilation–perfusion (V/Q) ratio. In other words, the dorsal areas of the lungs, often involved in inflammatory processes, are no longer compressed by the mediastinal and abdominal structures, leading to a more efficient gas exchange [6,7]. Prone positioning is also recommended by the Surviving Sepsis Campaign COVID-19 guidelines in moderate or severe COVID-19-related ARDS [8]. Since the beginning of the pandemic, numerous studies have shown the feasibility of pronation in non-intubated patients and have reported an improvement in the oxygenation and respiratory rates during prone positioning, in addition to the use of a high flow nasal cannula (HFNC) and non-invasive ventilation (NIV) in COVID-19 [4,9]. A recent systematic review and meta-analysis concluded that in patients with COVID-19-related acute hypoxemic respiratory failure, awake prone positioning reduced the need for intubation, particularly among those requiring advanced respiratory support as well as among those in ICU settings [10]. The rationale for awake prone positioning consists in the possibility of reducing the patient’s respiratory efforts and eventually the risk of a self-induced lung injury (P-SILI) [11]. Similarly to ARDS and observed in COVID-19 related ARDS, the parenchymal opacities are bilateral with peripheral, posterior and basal predominancies [12]. In classic ARDS, Papazian et al. did not find a correlation between the CT-scan pattern and prone position response in terms of a PaO_2_/FiO_2_ ratio improvement [13]. However, those patients were intubated, sedated and not diagnosed with COVID-19 related ARDS. Therefore, in severe COVID-19, the hypothesis that the more opacities in the dependent regions, the better the oxygenation can be achieved during prone positioning, is worth investigating.

The aim of the present study was to investigate whether the amount, patterns and distribution of lung abnormalities evaluated by the baseline chest CT-scan could predict an improvement in oxygenation in awake prone-positioned COVID-19 related ARDS patients.

## 2. Materials and Methods

### 2.1. Study Design, Participants and Treatment Protocol

This was a retrospective monocentric study approved by the local Ethics Committee (Comitato Etico di Bergamo, Italy: protocol N°97/2021). Verbal consent was obtained when feasible, according to local protocol. Written consent was not collected in order to avoid contamination from the paper, in accordance with hospital dispositions at that time. We included adult patients with a SARS-CoV-2 infection confirmed by a laboratory test (namely, a molecular PCR swab) who were hospitalized at Papa Giovanni XXIII Hospital (Bergamo, Italy) during the second pandemic wave (October 2020–January 2021) and who were admitted to the sub-intensive respiratory unit. According to our internal protocol at that time, patients were admitted to the sub-intensive respiratory unit only after the failure of a CPAP trial (i.e., a PaO_2_/FiO_2_ < 200, a deterioration in the PaO_2_/FiO_2_ over time or a respiratory rate >30 breaths/min). All of the patients had a chest CT-scan before admission to the sub-intensive respiratory unit. An arterial line was placed in order to obtain a serial arterial blood gas analysis (ABG) and patients underwent a NIV trial with pronation. Sedation was used in cases of discomfort or agitation, usually with dexmedetomidine or morphine, with a Richmond Agitation Sedation Scale (RASS) target of 0/−1. The patients were encouraged to maintain the prone position for at least 3 h. According to our protocol, the respiratory rate and ABG were recorded immediately before a postural change (time point SP1), then 30 min to one hour after prone positioning (time point PP) and 30 min to one hour after resupination (timepoint SP2). We excluded from the present analysis data about patients with a chronic pulmonary disease requiring home non-invasive ventilation or oxygen therapy.

### 2.2. CT Image Acquisition and Quantitative Analysis

Chest CT-scans were performed using a standard dose protocol, either without or with a contrast intravenous injection, in the supine position and full inspiration, from the bases of the lungs to the apex, with two different 64-slice scanners (Brilliance, Philips, Amsterdam, The Netherlands; CT evolution, GE Healthcare, Chicago, IL, USA). The images were reconstructed with a 0.9–1.5 mm increment and using a sharp reconstruction kernel for the parenchyma. The DICOM data were transferred into a picture archiving and communication (PACS) workstation, and the CT images were evaluated separately by two radiologists using dedicated computer software (Thoracic VCAR software, GE Healthcare, North Richland Hills, TX, USA). Thoracic VCAR software was originally designed to quantify pulmonary emphysema in patients with Chronic Obstructive Pulmonary Disease (COPD). The software provides automatic segmentation of the lungs and permits a voxel classification based on Hounsfield Units’ (HU) values. During the COVID-19 pandemic, its use has been revised and adapted in order to permit a quantitative assessment of the lung involvement, in terms of percentage and volume. A colorimetric map was used to describe the different patterns of lung parenchyma abnormalities: emphysema (−1024/−977 HU; blue), healthy residual lung parenchyma (−976/−703 HU; yellow), the ground glass opacity (GGO) area (−702/−368 HU; pink) and the consolidations (−100/+5 HU; red). All parameters were also assessed in terms of distribution within the zones of the lung. Of note, the lung parenchyma was divided into two fields (i.e., anterior and posterior) by a plane passing through the bronchial branch for the apico–dorsal segment of the left upper lobe and for the apical segment of the right upper lobe. Then, the volumes (%) for both lungs were calculated. Finally, using the colorimetric map and the calculated lung volumes, we derived the volume occupied by each pattern in each different area of the lungs. Then, we expressed these volumes in the percentage related to the total lung volume and performed the statistical analysis. The eventual pulmonary thromboembolism was annotated but not included in the final analysis.

### 2.3. Statistical Analysis

Descriptive statistics were used to summarize the baseline characteristics of the patients. Continuous variables were expressed as mean and standard deviations (SD) or as median and interquartile ranges [IQR]. The comparison of continuous variables was conducted using the Student’s t test for variables with a normal distribution and using the Mann–Whitney U test for variables with a non-normal distribution. The categorical variables were expressed as absolute counts and percentages and were analyzed with the chi-square test or Fisher’s exact test. The correlation was assessed using Pearson’s correlation coefficient. All reported *p* values are two sided, and a *p* value < 0.05 was considered significant. The statistical analysis was performed using SPSS 27.0 (SPSS, Inc., Chicago, IL, USA).

### 2.4. Outcomes

The primary outcome was the evaluation of the relationship between the parenchymal abnormalities’ distribution in the baseline CT-scan (i.e., posterior/anterior ratio) and the improvement in oxygenation after prone positioning (i.e., a SP2 PaO_2_/FiO_2_ ratio > SP1 PaO_2_/FiO_2_ ratio). Responders were defined as patients with PaO_2_/FiO_2_ SP2 > SP1. However, since there is no validated definition for a prone position responder, we carried out a supplemental analysis (see Supplementary file) using two different definitions, as other authors had previously carried out (i.e., 10 and 20% increases in PaO_2_/FiO_2_ when compared with SP1) [10]. To better investigate our topic of interest, the correlation between the distribution of the parenchymal abnormalities (expressed as, i.e., the ratio between the percentage of the parenchymal involvement of the posterior and anterior regions, with 1 = homogeneous distribution, <1 = predominant anterior and >1 = predominant dorsal involvement) and the changes in the oxygenation between timepoints (i.e., the PaO_2_/FiO_2_ ratio before and after prone positioning: the oxygenation results after supine positioning SP2, minus the results from supine positioning SP1 and prone positioning) has been evaluated.

## 3. Results

Forty-five patients with COVID-19 related ARDS were included in the current study. Their demographic and clinical characteristics are shown in Table 1. Patients were mainly male (73%), with a median age of 64 years. Chronic comorbidities were frequent, as indicated by a median Charlson Comorbidity Index (CCI) of 4. The median time between the onset of symptoms and arrival to the Emergency Room (ER) was 6 days, and the patients were admitted to the sub-intensive respiratory unit on average one day after hospitalization. Two patients (4%) had pulmonary thromboembolism upon admission. Baseline blood tests showed an activated inflammatory response (median C-reactive protein of 8.1 mg/dl and median D-dimer of 682 ng/mL).

### 3.1. Chest CT Parenchymal Abnormalities

**Figure 1:**
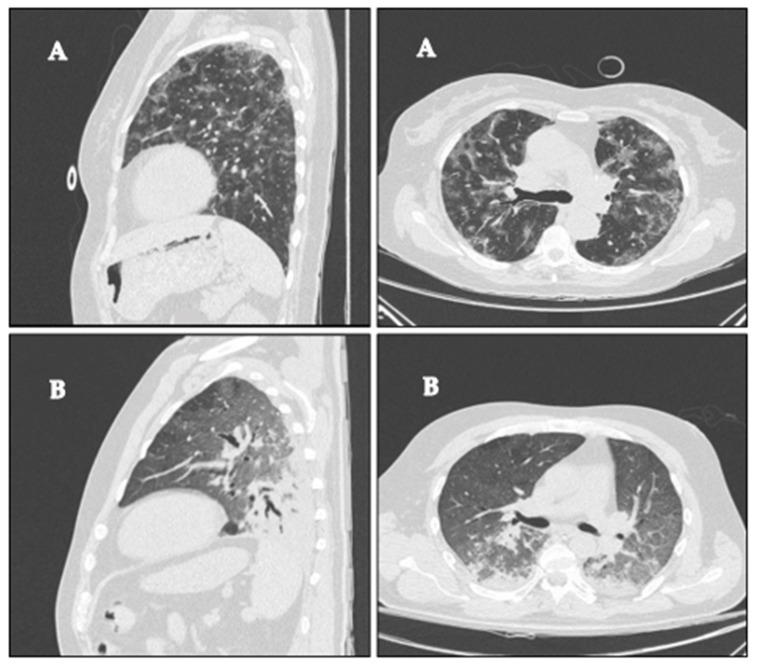
COVID-19 representative sagittal and axial computer tomography scans of two patients with (**A**) homogeneous distribution of the disease (ratio between the percentage of the parenchymal involvement of posterior and anterior regions of 0.95), PaO_2_/FiO_2_ of 94, 280 and 137 at SP1, PP and SP2, respectively, and (**B**) prevalent dorsal distribution of the disease (ratio between the percentage of the parenchymal involvement in the posterior and anterior regions of 2.92), PaO_2_/FiO_2_ of 141, 166 and 154 at SP1, PP and SP2, respectively.

Emphysema was negligible, involving on average much less than 1% of the lungs (Table 2). On average, 50% of the overall lung volume was affected by the disease. The most common parenchymal abnormality was GGO, followed by consolidation (44% and 4%, respectively, Table 2). As expected, the affected parenchyma was prevalently basal, accounting on average for 40% of the superior zones and 56% of the basal ones. The parenchymal abnormalities were predominantly posterior, as demonstrated by a posterior/anterior ratio of GGO and consolidation of 1.7. This heterogeneous distribution was consistent with all parenchymal abnormalities, with posterior/anterior ratios of 1.5 and 4.4 for ground glass opacity and consolidation, respectively (Table 2). Figure 1 shows two representative patients with different distributions of parenchymal abnormalities.

### 3.2. Response to Prone Position and Correlation with Imaging

**Figure 2:**
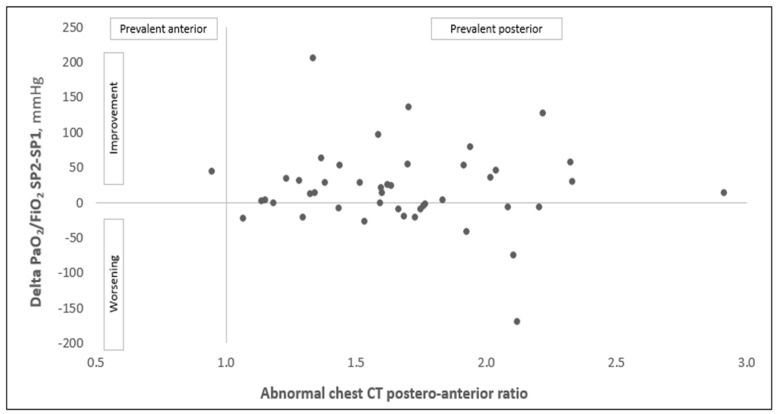
Correlation between the distribution of the parenchymal abnormalities (i.e., ratio between the percentage of the parenchymal involvement of the posterior and anterior regions, with 1 = homogeneous distribution, <1 = predominant anterior and >1 = predominant dorsal involvement) and changes in the PaO_2_/FiO_2_ ratio before and after prone positioning (oxygenation results from supine positioning SP2 minus supine positioning SP1 after prone positioning). Results of Pearson’s correlation test: R = 0.094, *p* = 0.526.

Oxygenation improved on average by 67% (change of PaO_2_/FiO_2_: +98) during prone positioning (Table 1). Once supine positioning was resumed, the improvement in oxygenation was maintained in 28 patients (62% of the overall population, categorized as “responders”). Table 1 and Table 2 show comparisons of the clinical and radiological features between responders and non-responders. This comparison did not show significant differences in terms of the demographic, clinical features and blood test analysis, with the only exception being the amount of time between hospitalization and admission to the sub-intensive care unit, which resulted in a marginally longer period of time in responders (2 [1;4] vs. 1 [0;2] days in responders and non-responders, respectively, *p* = 0.05). The same analysis did not show a significant difference between responders and non-responders in terms of the amount, patterns and distribution of the parenchymal abnormalities in the chest CT. Notably, the two groups showed comparable amounts of abnormal parenchyma (51 and 49%, for responders and non-responders, respectively, *p* = 0.78), with the same predominantly posterior distribution (posterior/anterior ratio of the ground glass plus the consolidation of 1.70 in both groups, *p* = 0.72). We obtained similar results (i.e., no difference between responders and non-responders for all of the variables in Table 1 and Table 2) by changing the definition of responders as patients with a 10% and 20% increase in their PaO_2_/FiO_2_ ratio between time points SP1 and SP2 (see Supplementary Data: Tables S1–S4). The correlation between the distribution of parenchymal abnormalities and the changes in oxygenation between timepoints SP1 and SP2 is shown in Figure 2. The correlation was assessed using Pearson’s correlation test. The analysis did not show any linear correlation between the two variables (R = 0.094, *p* = 0.526).

## 4. Discussion

The main findings of our study can be summarized as follows: (1) the radiological involvement at admission in COVID-19 patients who require NIV can be extensive, on average up to 50% of the lung parenchyma, mainly with GGO and dorsal involvement; (2) in about 60% of patients, the PaO_2_/FiO_2_ ratio evaluated at resupination is improved in absolute terms compared to the baseline; (3) the amount, pattern and distribution of lung abnormalities evaluated by CT-scans at admission do not predict the response for awake prone positioning evaluated by the PaO_2_/FiO_2_ ratio.

Focusing on the radiological findings, we found a greater prevalence of the ground glass pattern and involvement of the dorsal-basal regions in the CT-scans, as already extensively described in COVID-19 related ARDS [14,15,16]. In intubated patients with non-COVID-19 related ARDS, several studies have shown that prone positioning improves oxygenation, contributes to preventing ventilator-induced lung injury (VILI) and reduces 28 and 90-day mortality in patients with severe hypoxemia [5].

The PaO_2_/FiO_2_ ratio improvement during pronation is generally attributed to several phenomena. Among these, we can mention the fluids’ migration to ventral zones due to gravity resulting in a better expansion of the dorsal areas, that are free to reopen without the weight of the mediastinal structures [5,17,18]. Of note, in non-CARDS, the distribution of perfusion is similar in the prone and supine positions; therefore, optimizing ventilation of the dorsal areas results in an optimization of the ventilation-perfusion matching [19,20,21,22]. In non-COVID-19 related ARDS, the literature about the relationship between the baseline CT-scan pattern distribution and the response to the pronation in terms of the PaO_2_/FiO_2_ variations is limited. In 2002, Papazian et al. hypothesized that the more extensive consolidations in the CT-scans, the greater the PaO_2_/FiO_2_ improvement. In their research, despite thirty-one of the enrolled ARDS patients experiencing an improvement of at least 33% in the PaO_2_/FiO_2_ ratio from the baseline (67% of the cohort), the CT-scan abnormalities could not predict a response to pronation [13].

However, COVID-19 related ARDS seems to differ somehow from typical ARDS [23]. In the context of COVID-19 related ARDS, patients present with highly variable patho-physiological characteristics, in spite of a similar degree of hypoxemia [24,25]. Of note, in the context of COVID-19 related ARDS, it has been shown that “awake” pronation is feasible (i.e., maintainable for at least 3 h/day) and effective in rapidly ameliorating blood oxygenation in patients with COVID-19-related ARDS. Similarly to our results, Coppo et al. described a PaO_2_/FiO_2_ ratio improvement at one hour after resupination in 50% of their cohort [9].

With regard to the evaluation of CT-scan images and the pronation in COVID-19 related ARDS, Rossi et al. described how the response to prone positioning and the recruitment decreased in time due to the progressive lung consolidation versus atelectasis [26]. Our research does not find an association between the pathological CT-scan patterns and the distribution at admission and the response in terms of the PaO_2_/FiO_2_ variations. The comprehension of the pathophysiological mechanisms responsible for these results is outside the scope of the present study. However, it is possible to hypothesize that improvements in the PaO_2_/FiO_2_ ratio during and after pronation may be due to better V/Q coupling. During the early stages of COVID-19 related ARDS, when the prevailing patterns are the peripherical ground glass opacities rather than the consolidation or atelectasia, there is little recruitment of the consolidated areas. In this context, Busana et al. have developed a mathematical model that shows how hypoxia depends more on the hyperperfusion of poorly ventilated lung regions rather than from a shunt of the consolidated regions [27]. Indeed, the consolidation pattern accounted for less than 5% of the overall lung parenchyma in our cohort. In other words, both in non-COVID-19 related ARDS and COVID-19 related ARDS, the PaO_2_/FiO_2_ improvement with pronation could be attributable to better coupling of ventilation and perfusion. However, in the former scenario, it is mainly due to a ventilatory optimization, while in the latter, the perfusion redistribution could be the determinant of a gas exchange amelioration.

This study has some limitations. First, it is a single-center retrospective study without a priori evaluation of the sample size. However, the number of enrolled patients is in line with previous studies [14]. Furthermore, considering the total absence of any trends in the differences or correlations, we can reasonably rely on our conclusions. Second, there is not an accepted PaO_2_/FiO_2_ cut-off ratio to identify responders and non-responders; therefore, we opted for an absolute variation of this parameter and this choice can be criticized. However, this approach has already been used in another similar study [9]; moreover, when conducting additional analyzes with a 10% or 20% improvement cut-off, we were unable to obtain different results (Supplementary Data: Tables S2 and S4). Third, we limited our evaluation to the first cycle of pronation. However, in a different model (i.e., intubated patients under mechanical ventilation), a recent study demonstrated that a difference in the gas-exchange improvement is possible between the first and the subsequent cycles of pronation [28]. Finally, we evaluated patients admitted to the sub-intensive respiratory unit after the failure of a CPAP trial without differentiating patients in the early or late phase of the disease.

## 5. Conclusions

In conclusion, our study demonstrates that the extent, distribution and pattern of abnormalities in chest CT-scans at admission are not associated with the response to pronation in terms of variation in the PaO_2_/FiO_2_ ratio in COVID-19 related ARDS. Therefore, physicians dealing with this category of patients should not rely on imaging at presentation when evaluating whether to pronate patients.

## Figures and Tables

**Table 1:** Demographic and clinical features.

	All Patients (*n* = 45)	Responders * (*n* = 28)	Non-Responders * (*n* = 17)	*p*-Value
**Male gender**, *n* (%)	33 (73)	20 (71)	13 (76)	0.72
**Age**, years	63 (12)	63 (11)	65 (14)	0.58
**BMI**, Kg/m^2^	27.3 (3.7)	27.8 (3.1)	26.5 (4.5)	0.27
**Interval: Symptoms–Hospital Admission**, days	6 [4;8]	7 [4;9]	5 [3;7]	0.33
**Interval: Hospital Admission–SubICU**, days	1 [1;4]	2 [1;4]	1 [0;2]	0.05
**NIV Duration**, days	6 [4;9]	7 [4;10]	5 [2;9]	0.11
**PEEP**, cmH_2_O	8 [6;9]	8 [6;8]	8 [7;10]	0.27
**Pressure Support**, cmH_2_O	8 [6;10]	8 [6;9]	8 [6;10]	0.50
**Charlson Index**	4 [3;5]	4 [2;5]	4 [3;6]	0.48
**Hemoglobin**, g/dL	14.0 [12.5;14.8]	14.1 [12.9;14.7]	13.9 [12.3;15.4]	0.93
**Platelets**, *10^^9^/L	206 [159;272]	204 [161;266]	227 [151;288]	0.62
**LDH**, U/L	399 [311;468]	399 [215;462]	404 [302;484]	0.82
**C-Reactive Protein**, mg/dL	8.1 [4.5;15.8]	9.0 [5.3;16.4]	6.9 [4.2;15.0]	0.31
**D-Dimer**, ng/mL	682 [506;1099]	657 [499;843]	695 [507;2059]	0.22
**Pulmonary thromboembolism**, *n* (%)	2 (4)	2 (7)	0 (0)	0.26
**Supine position before prone position**				
**Respiratory rate**, acts/min	20 [18;25]	21 [18;24]	19 [17;25]	0.68
**pH**	7.45 [7.43;7.47]	7.44 [7.42;7.47]	7.45 [7.43;7.49]	0.52
**PaCO_2_**, mmHg	35.0 (3.8)	34.8 (4.2)	35.4 (3.1)	0.63
**HCO_3_**^−^, mmol/L	25.0 [24.0;27.1]	25.1 [24.2;26.8]	24.6 [23.9;29.6]	0.54
**PaO_2_/FiO_2_**	140 [108;169]	134 [108;155]	145 [108;255]	0.28
**Prone position**				
**Respiratory rate**, acts/min	19 (4)	20 (5)	19 (5)	0.67
**PaO_2_/FiO_2_**	246 (105)	262 (80)	220 (135)	0.26
**Supine position after prone position**				
**Respiratory rate**, acts/min	20 [18;25]	21 [17;25]	20 [17;26]	0.87
**PaO_2_/FiO_2_**, mmHg/%	157 [111;198]	167 [137;200]	112 [95;193]	0.01
**Delta**				
**PaO_2_/FiO_2_ PP–SP1**	98 (84)	127 (74)	50 (78)	<0.01
**PaO_2_/FiO_2_ PP1–SP1, %**	67 [21;113]	92 [35;130]	19 [−6;69]	<0.01
**PaO_2_/FiO_2_ SP2–SP1**	13 [−9;44]	32 [15;55]	−10 [−26;−6]	<0.01
**PaO_2_/FiO_2_ SP2–SP1, %**	9 [−6;36]	24 [13;51]	−8 [−19;−4]	<0.01

(* Responders: PaO_2_/FiO_2_ S2 > S1). Data are reported as mean (standard deviation) or median [interquartile], according to their distribution. SP1: supine positioning before postural change; PP: prone positioning; SP2: supine positioning after resupination. Significant differences in bold.

**Table 2:** Chest CT parenchymal abnormalities and distribution.

	All Patients (*n* = 45)	Responders * (*n* = 28)	Non-Responders * (*n* = 17)	*p*-Value
**OVERALL**				
**Healthy parenchyma**	50 (17)	51 (17)	49 (16)	0.78
**Emphysema**	0.02 [0.00;0.08]	0.00 [0.00;0.07]	0.03 [0.01;0.19]	0.06
**Ground glass**	44 (14)	43 (13)	46 (15)	0.61
**Consolidation**	4 [2;9]	4 [2;10]	5 [2;7]	0.98
**Ground glass + consolidation**	51 (16)	50 (17)	51 (16)	0.92
**ANTERIOR**				
**Healthy parenchyma**	61 (15)	61 (15)	61 (15)	0.98
**Emphysema**	0.01 [0.00;0.09]	0.01 [0.00;0.04]	0.05 [0.00;0.21]	0.16
**Ground glass**	37 (14)	36 (13)	37 (15)	0.80
**Consolidation**	1 [0;2]	1 [1;3]	1 [1;2]	0.85
**Ground glass + consolidation**	39 (15)	39 (15)	39 (15)	0.99
**POSTERIOR**				
**Healthy parenchyma**	38 (18)	39 (19)	37 (17)	0.70
**Emphysema**	0.00 [0.00;0.05]	0.00 [0.00;0.01]	0.02 [0.00;0.09]	0.07
**Ground glass**	51 (16)	50 (15)	54 (17)	0.43
**Consolidation**	7 [3;15]	7 [3;15]	8 [4;13]	1.00
**Ground glass + consolidation**	62 (18)	61 (19)	63 (17)	0.76
**POSTERIOR/ANTERIOR RATIO**				
**Healthy parenchyma**	0.6 (0.2)	0.6 (0.2)	0.6 (0.2)	0.65
**Ground glass**	1.5 (0.4)	1.5 (0.4)	1.5 (0.4)	0.76
**Consolidation**	4.4 [2.5;6.4]	4.3 [2.2;6.2]	4.5 [2.5;8.6]	0.66
**Ground glass + consolidation**	1.7 (0.4)	1.7 (0.4)	1.7 (0,3)	0.72

(* Responders: PaO_2_/FiO_2_ S2 > S1). Data indicate the percentage, if not otherwise specified, and are expressed as mean (standard deviation) or median [interquartile] according to their distribution.

## Data Availability

The datasets generated and analyzed during the current study are available in the Papa Giovanni XXIII Hospital digital repository. The datasets generated and analyzed during the current study are not publicly available due individual privacy policy. However, they are available from the corresponding author upon reasonable request.

## Supplementary Materials

The following supporting information can be downloaded at https://www.mdpi.com/article/10.3390/diagnostics12081848/s1. Supplementary File: included Tables S1–S4. Table S1. Demographic and clinical features of the population of the study (considering as responders: PaO_2_/FiO_2_ S2 > S1 more than 10%). Table S2. Chest CT parenchymal abnormalities and distribution (considering as responders: PaO_2_/FiO_2_ S2 > S1 more than 10%). Table S3. Demographic and clinical features (considering as responders: PaO_2_/FiO_2_ S2 > S1 more than 20%). Table S4. Chest CT parenchymal abnormalities and distribution (considering as responders: PaO_2_/FiO_2_ S2 > S1 more than 20%).
