# Supplementary material for: Extent and Distribution of Parenchymal Abnormalities in Baseline CT-Scans Do Not Predict Awake Prone Positioning Response in COVID-19 Related ARDS
Source: Diagnostics (Basel). 2022 Jul 30;12(8):1848. doi: 10.3390/diagnostics12081848 (PMC9406535; doi:10.3390/diagnostics12081848)
Supplement: Supplementary file 1 [file diagnostics-12-01848-s001.zip › diagnostics-1822351-supplementary.pdf]

diagnostics

MDPI

Supplementary

Table S1. Demographic and clinical features (*Responders: PaO_2/FiO_2 S2>S1 more than 10%). Data are reported as mean (standard deviation) or median [interquartile] according to their distribution. SP1: supine position before postural change; PP: prone position; SP2: supine position after resupination. Significant difference in bold.

	**All patients (*n* = 45)**	**Responders* (*n* = 22)**	**Non responders* (*n* = 23)**	***p*-value**
**Male gender,** *n* (%)	33 (73)	15 (68)	18 (78)	0.45
**Age,** *years*	63 (12)	63 (10)	65 (14)	0.67
**BMI,** Kg/m^2	27.3 (3.7)	28.1 (3.0)	26.6 (4.2)	0.17
**Interval Symptoms-Hospital Admission,** *days*	6 [4;8]	7 [4;9]	6 [4;7]	0.44
**Interval Hospital Admission-SubICU,** *days*	1 [1;4]	3 [1;4]	1 [0;2]	**0.02**
**NIV Duration,** *days*	6 [4;9]	8 [4;11]	5 [2;8]	0.14
**PEEP,** *cmH$_2$O*	8 [6;9]	8 [6;8]	8 [7;9]	0.32
**Pressure Support,** *cmH$_2$O*	8 [6;10]	8 [6;9]	8 [6;10]	0.78
**Charlson Index**	4 [3;5]	4 [2;5]	4 [3;6]	0.44
**Hemoglobin,** *g/dL*	14.0 [12.5;14.8]	13.9 [12.9;14.5]	14.1 [12.4;15.4]	0.59
**Platelets,** **10^^{9}/L*	206 [159;272]	198 [156;253]	227 [159;289]	0.30
**LDH,** *U/L*	399 [311;468]	378 [293;466]	407 [314;480]	0.37
**C-Reactive Protein,** *mg/dL*	8.1 [4.5;15.8]	8.1 [5.1;16.1]	8.1 [4.4;15.8]	0.77
**D-Dimer,** *ng/mL*	682 [506;1099]	608 [504;865]	695 [507;1333]	0.41
**Pulmunary trombo-embolysm,** *n (%)*	2 (4)	1 (5)	1 (4)	0.97
**Supine position before prone position**				
**Respiratory rate,** *acts/min*	20 [18;25]	20 [18;22]	20 [18;25]	0.47
**pH**	7.45 [7.43;7.47]	7.45 [7.43;7.47]	7.45 [7.42;7.47]	0.77
**$PaCO_2$,** *mmHg*	35.0 (3.8)	35.0 (3.9)	35.0 (3.8)	0.98
**HCO_3^-,** *mmol/L*	25.0 [24.0;27.1]	25.1 [24.5;27.1]	24.6 [23.9;29.0]	0.87
**PaO_2/FiO_2**	140 [108;169]	134 [114;158]	141 [107;181]	0.59
**Prone position**				
**Respiratory rate,** *acts/min*	19 (4)	19 (5)	19 (4)	0.80
**PaO_2/FiO_2**	246 (105)	278 (80)	216 (119)	**0.05**
**Supine position after prone position**				
**Respiratory rate,** *acts/min*	20 [18;25]	20 [16;23]	20 [18;25]	0.30
**PaO_2/FiO_2,** *mmHg/%*	157 [111;198]	188 [149;245]	112 [102;165]	**<0.01**
**Delta**				
**PaO_2/FiO_2 PP-SP1**	98 (84)	140 (74)	57 (73)	**<0.01**
**PaO_2/FiO_2 PP1-SP1, %**	67 [21;113]	105 [42;137]	23 [−5;77]	**<0.01**
**PaO_2/FiO_2 SP2-SP1**	13 [−9;44]	44 [28;67]	−8 [−22;2]	**<0.01**
**PaO_2/FiO_2 SP2-SP1, %**	9 [−6 ;36]	36 [19;64]	−4 [−16;2]	**<0.01**

Table S2. Chest CT parenchymal abnormalities and distribution (*Responders: PaO_2/FiO_2 S2>S1 more than 10%). Data indicates the percentage, if not otherwise specified, and are expressed as mean (standard deviation) or median [interquartile] according to their distribution. Significant difference in bold.

	All patients (n = 45)	Responders* (n = 22)	Non responders* (n = 23)	p-value
**OVERALL**				
**Healthy parenchyma**	50 (17)	52 (15)	48 (18)	0.36
**Emphysema**	0.02 [0.00;0.08]	0.00 [0.00;0.07]	0.03 [0.00;0.15]	0.14
**Ground glass**	44 (14)	42 (12)	47 (15)	0.23
**Consolidation**	4 [2;9]	4 [2;9]	5 [2;8]	0.63
**Groung glass + consolidation**	51 (16)	48 (15)	54 (17)	0.23
**ANTERIOR**				
**Healthy parenchyma**	61 (15)	63 (14)	58 (16)	0.25
**Emphysema**	0.01 [0.00;0.09]	0.01 [0.00;0.04]	0.02 [0.00;0.13]	0.33
**Ground glass**	37 (14)	34 (13)	39 (15)	0.29
**Consolidation**	1 [0;2]	1 [1;3]	1 [1;2]	0.98
**Groung glass + consolidation**	39 (15)	36 (14)	41 (16)	0.29
**POSTERIOR**				
**Healthy parenchyma**	38 (18)	42 (18)	34 (18)	0.14
**Emphysema**	0.00 [0.00;0.05]	0.00 [0.00;0.01]	0.02 [0.00;0.06]	**0.02**
**Ground glass**	51 (16)	48 (13)	54 (17)	0.21
**Consolidation**	7 [3;15]	7 [3;15]	8 [4;17]	0.60
**Groung glass + consolidation**	62 (18)	58 (18)	66 (18)	0.16
**POSTERIOR/ANTERIOR RATIO**				
**Healthy parenchyma**	0.6 (0.2)	0.6 (0.2)	0.6 (0.2)	0.21
**Ground glass**	1.5 (0.4)	1.5 (0.4)	1.5 (0.4)	0.80
**Consolidation**	4.4 [2.5;6.4]	4.3 [2.9;6.1]	4.5 [2.4;8.1]	0.62
**Groung glass + consolidation**	1.7 (0.4)	1.7 (0.4)	1.7 (0.4)	0.90

Table S3. Demographic and clinical features (*Responders: PaO_2/FiO_2 S2>S1 more than 20%). Data are reported as mean (standard deviation) or median [interquartile] according to their distribution. SP1: supine position before postural change; PP: prone position; SP2: supine position after resupination. Significant difference in bold.

	**All patients (*n* = 45)**	**Responders* (*n* = 16)**	**Non responders* (*n* = 29)**	***p*-value**
**Male gender,** *n* (%)	33 (73)	11 (69)	22 (76)	0.61
**Age,** *years*	63 (12)	64 (11)	64 (13)	0.34
**BMI**, Kg/m^2	27.3 (3.7)	28.0 (3.0)	27.0 (4.1)	0.70
**Interval Symptoms-Hospital Admission,** *days*	6 [4;8]	6 [3;7]	7 [4;9]	0.25
**Interval Hospital Admission-SubICU,** *days*	1 [1;4]	2 [1;4]	1 [0;4]	0.12
**NIV Duration,** *days*	6 [4;9]	8 [4;12]	5 [4;8]	0.17
**PEEP,** *cmH_2O*	8 [6;9]	7 [6;8]	8 [7;10]	0.07
**Pressure Support,** *cmH_2O*	8 [6;10]	8 [6;10]	8 [6;9]	0.34
**Charlson Index**	4 [3;5]	4 [3;5]	4 [3;6]	0.66
**Hemoglobin,** *g/dL*	14.0 [12.5;14.8]	13.9 [12.9;14.4]	14.1 [12.5;15.2]	0.60
**Platelets,** **10^9/L*	206 [159;272]	182 [128;239]	241 [168;290]	**0.02**
**LDH,** *U/L*	399 [311;468]	398 [324;470]	405 [302;469]	0.90
**C-Reactive Protein,** *mg/dL*	8.1 [4.5;15.8]	6.3 [4.6;14.3]	11.4 [4.5;16.2]	0.36
**D-Dimer,** *ng/mL*	682 [506;1099]	582 [499;843]	695 [507;1431]	0.33
**Pulmunary trombo-embolysm,** *n (%)*	2 (4)	1 (6)	1 (3)	0.66
**Supine position before prone position**				
**Respiratory rate,** *acts/min*	20 [18;25]	21 [18;24]	19 [17;25]	0.59
**pH**	7.45 [7.43;7.47]	7.46 [7.43;7.49]	7.44 [7.42;7.47]	0.48
**$PaCO_2$,** *mmHg*	35.0 (3.8)	35.0 (3.9)	35.0 (3.8)	0.94
**HCO_3^-,** *mmol/L*	25.0 [24.0;27.1]	25.3 [23.4;27.0]	25.0 [24.0;27.8]	0.90
**PaO_2/FiO_2**	140 [108;169]	127 [98;151]	145 [108;195]	0.13
**Prone position**				
**Respiratory rate,** *acts/min*	19 (4)	21 (5)	19 (4)	0.19
**PaO_2/FiO_2**	246 (105)	287 (76)	224 (113)	**0.03**
**Supine position after prone position**				
**Respiratory rate,** *acts/min*	20 [18;25]	21 [18;24]	20 [16;25]	0.46
**PaO_2/FiO_2,** *mmHg/%*	157 [111;198]	188 [142;238]	128 [104;182]	**0.02**
**Delta**				
**PaO_2/FiO_2 PP-SP1**	98 (84)	161 (70)	63 (70)	**<0.01**
**PaO_2/FiO_2 PP1-SP1, %**	67 [21;113]	118 [93;165]	31 [10;73]	**<0.01**
**PaO_2/FiO_2 SP2-SP1**	13 [−9;44]	53 [31;92]	−4 [−21;13]	**<0.01**
**PaO_2/FiO_2 SP2-SP1, %**	9 [−6 ;36]	47 [32;69]	−2 [−13;9]	**<0.01**

Table S4. Chest CT parenchymal abnormalities and distribution (*Responders: PaO_2/FiO_2 S2>S1 more than 20%). Data indicates the percentage, if not otherwise specified, and are expressed as mean (standard deviation) or median [interquartile] according to their distribution. Significant difference in bold.

	All patients (*n* = 45)	Responders* (*n* = 16)	Non responders* (*n* = 29)	*p*-value
**OVERALL**				
**Healthy parenchyma**	50 (17)	53 (15)	48 (18)	0.38
**Emphysema**	0.02 [0.00;0.08]	0.00 [0.00;0.07]	0.03 [0.00;0.09]	0.20
**Ground glass**	44 (14)	41 (11)	46 (15)	0.22
**Consolidation**	4 [2;9]	4 [2;9]	4 [2;9]	0.94
**Groung glass + consolidation**	51 (16)	47 (15)	53 (17)	0.27
**ANTERIOR**				
**Healthy parenchyma**	61 (15)	63 (14)	59 (16)	0.43
**Emphysema**	0.01 [0.00;0.09]	0.01 [0.00;0.03]	0.02 [0.00;0.10]	0.34
**Ground glass**	37 (14)	35 (13)	38 (15)	0.50
**Consolidation**	1 [0;2]	1 [1;2]	1 [1;2]	0.87
**Groung glass + consolidation**	39 (15)	37 (14)	40 (16)	0.48
**POSTERIOR**				
**Healthy parenchyma**	38 (18)	43 (17)	35 (19)	0.16
**Emphysema**	0.00 [0.00;0.05]	0.00 [0.00;0.00]	0.01 [0.00;0.06]	**0.04**
**Ground glass**	51 (16)	47 (11)	54 (17)	0.14
**Consolidation**	7 [3;15]	7 [4;14]	7 [3;17]	0.96
**Groung glass + consolidation**	62 (18)	57 (17)	64 (19)	0.18
**POSTERIOR/ANTERIOR RATIO**				
**Healthy parenchyma**	0.6 (0.2)	0.7 (0.2)	0.6 (0.2)	0.11
**Ground glass**	1.5 (0.4)	1.5 (0.4)	1.5 (0.4)	0.76
**Consolidation**	4.4 [2.5;6.4]	4.8 [3.3;6.5]	4 [2;7]	0.75
**Groung glass + consolidation**	1.7 (0.4)	1.6 (0.4)	1.7 (0.4)	0.63
